# Efficacy of alum for treatment of recurrent aphthous stomatitis

**Published:** 2016

**Authors:** Nasrin Rafieian, Hamidreza Abdolsamadi, Aliakbar Moghadamnia, Mina Jazayeri, Mohammadali Seif-Rabiee, Mina Salmanzadeh, Shahrbanoo Radi

**Affiliations:** 1 Department of Oral Medicine, Faculty of Dentistry, Alborz University of Medical Sciences, Karaj, Iran.; 2 Dental Research Center and Center for Molecular Medicine, Faculty of Dentistry, Alborz University of Medical Sciences, Karaj, Iran.; 3 Department of Pharmacology, Babol University of Medical Sciences, Babol, Iran.; 4 Department of Social Medicine, Hamedan University of Medical Sciences, Hamedan, Iran.; 5 Hamedan University of Medical Sciences, Hamedan, Iran.; 6 Faculty of Dentistry, Hamedan University of Medical Sciences, Hamedan, Iran.

**Keywords:** Aphthous, Stomatitis, Alum compounds

## Abstract

**Background:**

Recurrent aphthous stomatitis (RAS) is the most common painful ulcers of oral mucosal which can cause many sufferings. Treatment of RAS often includes administration of corticosteroids, analgesics and regulators of the immune system. However, considering the side effects of these medications, even their topical application must be done with caution. Alum is used in traditional medicine for treatment of oral ulcers without significant side effect. This study sought to assess the effect of topical application of alum on aphthous ulcers.

**Methods:**

This clinical randomized double-blind placebo-controlled study was conducted on 50 females aged 21 to 27 years. Mucosal adhesive patches were prepared in two forms of basic and 7% alum-containing patches. Subjects in two groups of case and control randomly received the mucosal adhesive patches containing alum and the basic patches, respectively three times in five days. Duration of recovery, changes in size of lesion and severity of pain were recorded. Data were entered into SPSS Version 16 and analyzed using t-test.

**Results:**

The average period of full recovery was 7.52 days in the case and 12.2 days in the control groups; which was significantly different (p<0.001). Size of wound and severity of pain were significantly lower at one, three and five days posttreatment compared to baseline values before treatment in the case group (p<0.001) and the difference in this regard between the case and control groups was statistically significant.

**Conclusion:**

Alum can significantly decrease the size of aphthous lesions, severity of pain and expedite the recovery of patients with RAS.

Aphthous lesions are acute, recurrent and painful ulcers in the non-keratinized mucosa (1). RAS is among the most common lesions of the oral mucosa in human beings involving 5-20% of the general population. However, its incidence varies from 5-50% in some ethnicities and different socioeconomic classes (2-4). Higher incidence rates (56%) have been reported among students and people of higher socioeconomic classes (4, 5). Also, its incidence is slightly higher in females and it occurs more commonly in individuals aged 10 to 40 years (4, 6). The main etiology of RAS is incompletely understood but appears to involve immune system dysfunction (7). The main factors currently known to be related to RAS include hereditary and genetic factors, hematologic defects, immunological disorders and local factors such as trauma and tobacco consumption (2, 8). One study reported the increased level of tumor necrosis factor (TNF) in association with RAS (4, 6). There is no definite treatment for RAS and supportive treatment is performed aiming to control pain, accelerate healing and prevent recurrence (6).

Supportive treatment usually includes administration of analgesics and regulators of the immune system. Topical treatments aim to accelerate recovery and relieve pain while systemic therapy is indicated for severe cases (9, 10). Corticosteroids are widely used to control aphthous lesions; however, even their topical application may be associated with some side effects. Recently, herbal medications have been suggested for treatment of these lesions due to having minimal or no side effects (4). Contemporary pharmaceutics pays special attention to local delivery of drugs to the target sites to decrease the side effects associated with systemic drug administration (11). 1% to 4% alum solution is used as mouthwash or gargle in the treatment of stomatitis and pharyngitis (12). 

Alum is found in the form of colorless, translucent and odorless crystals of KAI (SO4).12H2O (13). This compound has astringent and hemostatic properties when applied topically (14, 15). Alum causes contraction of tissues and enhances wound healing by decreasing the inflammation in the mucosal membrane (16). Since the normal flow of the saliva secreted from the salivary glands and oral movements may decrease or eliminate the effects of topical medications like suspensions, alum-containing mucosal adhesive patches were used in this study. The current study aimed to assess the effect of alum delivered via mucosal adhesive patches on recovery of RAS. 

## Methods

This experimental, double-blind clinical trial was approved by the Ethics Committee of Hamedan University of Medical Sciences and registered in IRCT (IRCT201305275678n2). The study was conducted on 50 female students in dormitories of Hamedan University of Medical Sciences in the age range of 21 to 27 years who referred to oral medicine department with complaint of multiple oral ulcers. The diagnosis of RAS was made based on its clinical appearance by specialist in oral medicine.

The inclusion criteria were history of RAS (at least monthly episodes of oral ulcer and most patients had continuous involvement.) and presence of early minor aphthous ulcer (not lasting more than three days of developing ulcers) .These lesions had to be in areas allowing easy use of mucosal adhesive patch. On the tongue, buccal mucosa, floor of the mouth, or labial mucosa. The sites of lesions in experimental groups were matched.

Exclusion criteria included: pregnancy or nursing, systemic conditions associated with aphthous ulcers such as Crown’s disease, Reiter’s syndrome and Behcet’s syndrome, cigarette smoking or use of chewing tobacco. Patients with fungal or viral infections under treatment with any medication and those taking non-steroidal anti-inflammatory drugs, acetaminophen, antihistamine or any other medications for treatment of aphthous ulcers were excluded from the study. Patients with fixed orthodontic appliances or retainers in close contact with aphthous ulcer were also excluded. Oral surgery in the past two weeks was also among the exclusion criteria. Written informed consent was obtained from the subjects. Patients who met the inclusion criteria received alum-containing mucosal adhesive patches or drug-free patches alternately (n=25 for the case and n=25 for the control group). 

**Figure 1:**
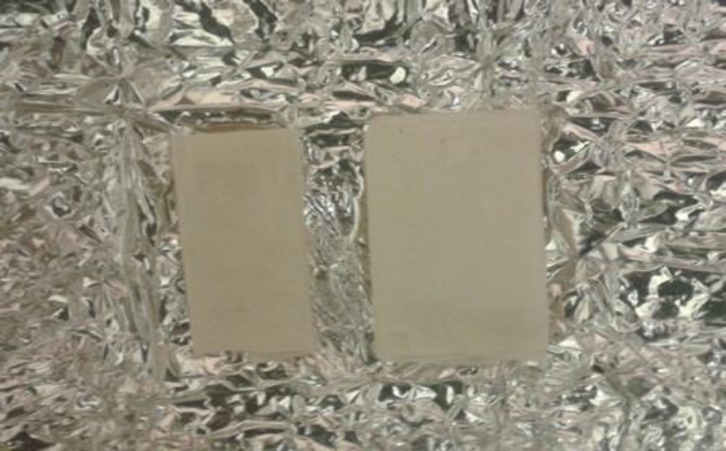
Mucosal adhesive

**Figure 2:**
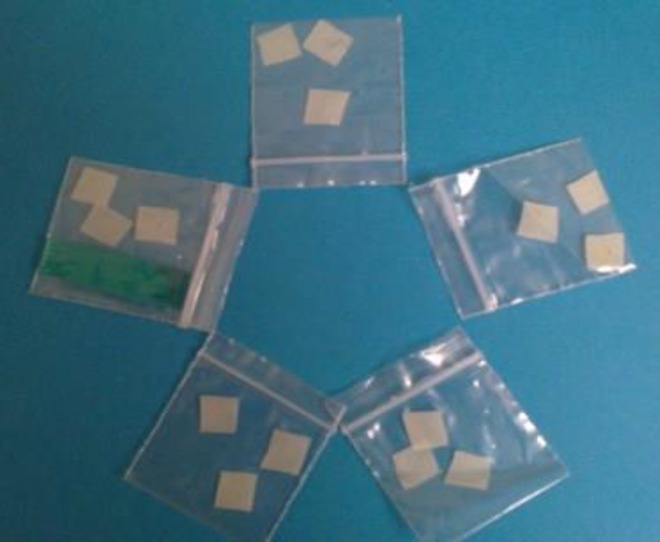
Packaging of mucosal adhesive patches

Mucosal adhesive patches measuring 1x1cm were prepared. Drug-free and aluminum-containing adhesive patches were placed into two different plastic bags. Each bag supplied three mucosal adhesive patches. Each subject participating in the study received five bags of the same type of mucosal adhesive patch along with a flexible 10mm ruler (figures 1-2). In the case group, mucosal adhesive patches contained 7% alum while in the control group, drug-free mucosal patches were used as placebo. Treatment was started from day three following the development of RAS, subjects used the patches as instructed for five days, three times daily at specific times (equal intervals) each time for 10 minutes. 

The patches had to be placed on the ulcer with dry hand and it was recommended not to move the tongue during its placement. Then the patient was reexamined by an examiner (oral medicine specialist) at third and fifth days. Data were recorded in a questionnaire. The questionnaires were filled out prior to treatment and at one, three and five days post-treatment. Recovery criteria included size of lesions, severity of pain and duration of recovery. A flexible, graded ruler was used to measure the size of lesions and the largest diameter of the lesion was recorded. The severity of pain was measured using visual analog scale (VAS). This scale comprised of a line measuring 100mm in length. The leftmost point indicated no pain and the rightmost point indicated most severe pain imaginable. So, in follow- up visits, degree of nonirritant pain of lesions was recorded by VAS scale.

Data were analyzed using SPSS 16 and t-test. Level of significance was set at p=0.05.

## Results

There were 25 female subjects in the case group with a mean age of 23.36±1.86 years and 25 in the control group with a mean age of 23.20±1.55 years. The mean age was not significantly different between the two groups (p=0.72). With regard to the localization of mucosal lesions, labial mucosa was the most common site of involvement (50% of cases) followed by buccal mucosa, tongue and floor of the mouth. Other areas had a lower number of lesions (2%) (table 1). The mean duration of recovery in case group was 7.52 days while in control group was 12.2. In this regard, duration of recovery in the treatment group was significantly lower (p= 0.000).

**Figure 3:**
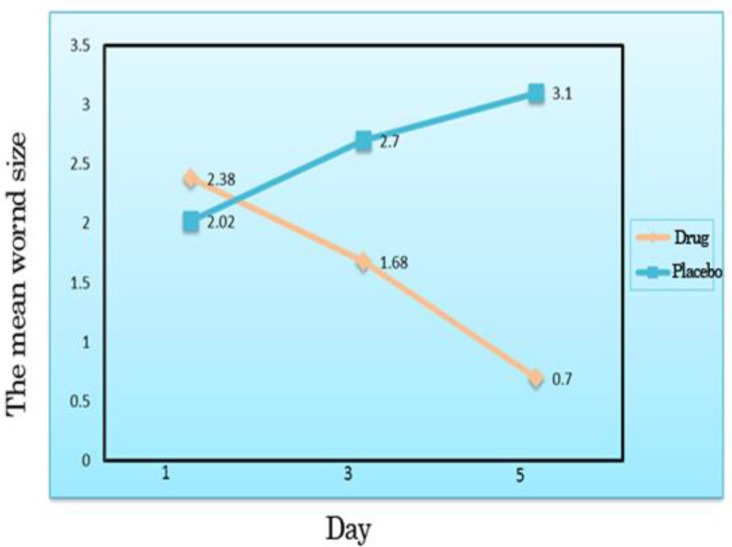
The mean wound size on one, three and five days posttreatment

The size of lesions at 1, 3 and 5 days was significantly lower in the case versus control group (p<0.001). Treatment with alum significantly decreased the mean diameter of the ulcer lesions in the case group compared to the controls at the same time points (table 2). In general, the mean wound diameter decreased in the case group at one, three and five days posttreatment; whereas, the size of ulcers increased at the same time points in the control group (figure 3). Severity of pain: the mean severity of pain at one, three and five days posttreatment decreased in both cases and controls compared to baseline pretreatment values (p<0.001). Alum caused significant pain reduction in posttreatment in the case compared to the control group (table 3).

**Table 1:** Frequency distribution of the site of aphthous lesions in subjects

**Site of RAS**	**Number**	**Frequency percentage**	**Percentage of cumulative frequency**
Tongue	8	16	16
Buccal mucosa	14	28	44
Floor of the mouth	2	4	48
Labial mucosa	25	50	98
Other	1	2	100
Total	50	100	

**Figure 4:**
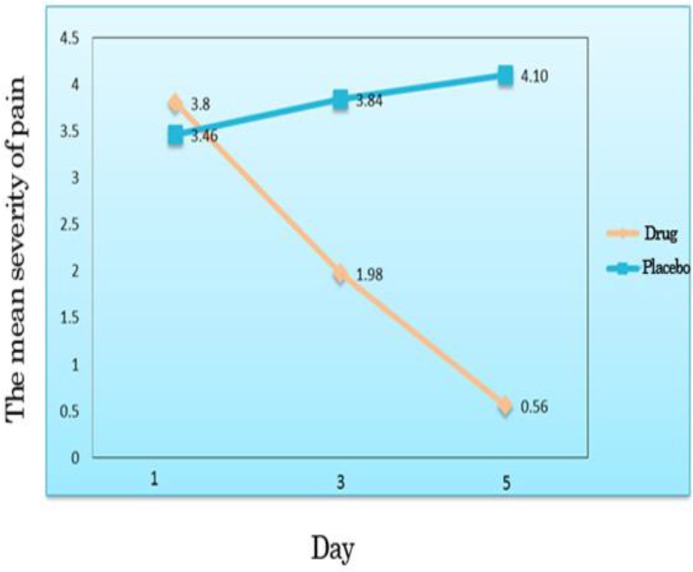
The mean severity of pain on one, three and five days posttreatment

As seen in figure 4, the mean severity of pain tended toward decrease versus the patients of the control group for whom pain tended toward increase.

**Table 2:** Comparison of the wound size (mm) on day one, three and five posttreatment compared to the baseline size in cases and controls

	**Group**	**Frequency**	**Mean change**	**SD**	**Pvalue**	**95% CI**	
						**Upper**	**Lower**
Difference in wound size on day one compared to baseline	Case Control	25 25	-0.4 0.54	0.92 0.45	0.0000	1.35	0.52
Difference in wound size on day three compared to baseline	Case Control	25 25	-1.10 1.22	1.36 0.81	0.0000	2.96	1.67
Difference in wound size on day five compared to baseline	Case Control	25 25	-2.8 1.62	1.57 1.64	0.0000	4.61	2.78

**Table 3:** Comparison of pain severity on day one, three and five posttreatment compared to baseline values in cases and controls

	**Group**	**Frequency**	**Mean change**	**SD**	**Pvalue**	**95% CI**	
						**Upper**	**Lower**
Difference in pain severity on day one compared to baseline	Case	25	48.1-	61.1	0.0000	10/3.	61.1
	Control	25	88.0	92.0			
Difference in pain severity on day three compared to baseline	Case	25	3.3-	22.2	0.0000	80.5	51.3
	Control	25	36.1	77.1			
Difference in pain severity on day five compared to baseline	Case Control	25 25	79.4- 1.68	97.2 2.8	0.0000	13.8	80.4

## Discussion

The available data indicate that most of aphthous lesions are mild and self-limiting, on the other hand, there is no curative therapy (16, 17). Therefore, the aim of this study was to assess the symptomatic treatment of RAS to prevent aphthous ulcers. Treatment are usually used for pain relief, maintaining function during attack and to reduce the severity of pain and recurrences of lesions (18, 19). A number of treatment options are only palliative such as benzocaine or diphenhydramine but others truly alter the course of the disease (14).

Topical treatments are preferred over systemic therapy due to fewer side effects. Topical treatment with mucosal adhesive patches such as Orabase, Zilactin, and cyanoacrylate have been shown to be useful (20, 21). In the this study, change in the size of ulcers and severity of pain compared to the baseline value in the case group was significantly different from that in the control group indicating efficacy of alum on decreasing the wound size and intensity of pain. Also this study showed that the mean duration of recovery was 7.52 and 12.2 days in the case and control groups, respectively. This difference was statistically significant and indicated significantly enhanced recovery of RAS with the use of alum. Altaei et al. in their study reported that 1, 3, 5 and 7% suspensions of alum had effect on healing of RAS compared to the control group. They reported changes in ulcer size and severity of pain (14). These results support our findings. Alum is found in nature and is commonly found in western areas of Iran. It has been recommended for treatment of RAS by Avicenna (12).Alum attaches to mucosal membrane proteins and decreases the permeability of cell membrane. Alum causes tissue contraction. 

It strengthens the matrix in the capillary endothelial walls and consequently prevents the passage of plasma proteins via the capillary walls and inhibits local edema. By decreasing the mucosal membrane inflammation, it accelerates wound healing as well. On the other hand, PH drops following the use of alum and helps the immune system for elimination of fatty acids produced by bacteria and thus, prevents bacterial growth and extension of lesions (16). Biocompatible adhesive patches were prepared in two forms of basic adhesive patches and the mucosal adhesive patches containing 7% alum in Babol School of Pharmacy. Moghadamnia et al. reported that the basic ingredients of biocompatible adhesive patches, similar to those used in the formulation of adhesive patches in the current study, caused no irritation in the oral mucosa of candidates and only two patients (13.3%) who used carboxymethyl cellulose adhesive patched complained of a tolerable salty taste (12). 

Delavarian et al. reported that sucralfate significantly expedited the recovery and decreased pain and size of aphthous lesions compared to the control group. Negatively-charged sucralfate molecules bond to positively-charged mucosal proteins and white blood cells. The main effect of this substance is its ability to locally increase the level of prostaglandins (22). 

Use of alum is associated with a risk of aluminum toxicity, previous studies reported no sign of oral mucosal injury following the use of mouthwash or alum mucosal adhesive patches and it was well-tolerated by patients (14). In the current study, no side effect was noticed due to the application of alum (16) and it was conveniently used by patients at home. There were no complaints of bad taste or burning sensation.

Altaei et al. (16) reported that in females, most lesions were on the tongue; which is in contrast to our finding. However, according to the Burket's Oral Medicine (2), RAS usually involves non-keratinized mucosa. Lip and buccal mucosa are often involved which is in line with our findings. In a review study, Shashy et al. (23) reported that minor aphthous ulcers commonly affected the free oral mucosa i.e. labial, buccal and lingual mucosa. According to a report by Al-Abbasi et al., (24) labial and buccal mucosa were the most common sites of involvement (25.9%). 

**Conclusion**

The results showed that alum significantly decreased the size of aphthous lesions and severity of pain and expedited the recovery of patients with RAS with lack of any important side effects. 

## Citation:

Rafieian N, Abdolsamadi H, Moghadamnia A, et al. Efficacy of alum for treatment of recurrent aphthous stomatitis. Caspian J Intern Med 2016; 7(3): 201-205.
